# Osler on Medical Women

**Published:** 1907-10

**Authors:** 


### Osler on Medical Women

Prof. Wm. Osler delivered the address at the London Medical School for women at their recent commencement. He did not say that all women practitioners should be chloroformed; in fact, he never said that of anybody. He told the graduates that many women were especially adapted to work on the highest problems of scientific medicine. He cited bacteriology, histology and even pathology as peculiarly suitable to them. Asylum work was another important line for their fitness. He would be glad to see one or two women physicians in every asylum. General practice was so hard for men that he was sorry for the woman who had to face it. As to restricting them to a special practice among women and children, which many would like to see, Osler remarked that women did not believe much in men, but that they believed less in women. This last remark was greeted with cheers when he added that this was one of the serious problems in connection with the progress of women. His advice to them was to so conduct their lives that others would have trust in them. He thought that the work of inspection of schools could be done better by women than by men. He thought that in the mission fields, especially in foreign or Mohammedan countries, there was room and to spare for women in medicine.

